# Supplementary material for: Seminoma and Embryonal Carcinoma Footprints Identified by Analysis of Integrated Genome-Wide Epigenetic and Expression Profiles of Germ Cell Cancer Cell Lines
Source: PLoS One. 2014 Jun 2;9(6):e98330. doi: 10.1371/journal.pone.0098330 (PMC4041891; doi:10.1371/journal.pone.0098330)
Supplement: File S1 — ZIP file containing DMRforPairs output for significant regions. Please start from the html files. (ZIP) [file pone.0098330.s008.zip › figures/5708.pdf]

RegionID: 5708, chr10:49812686−49813434−M_values

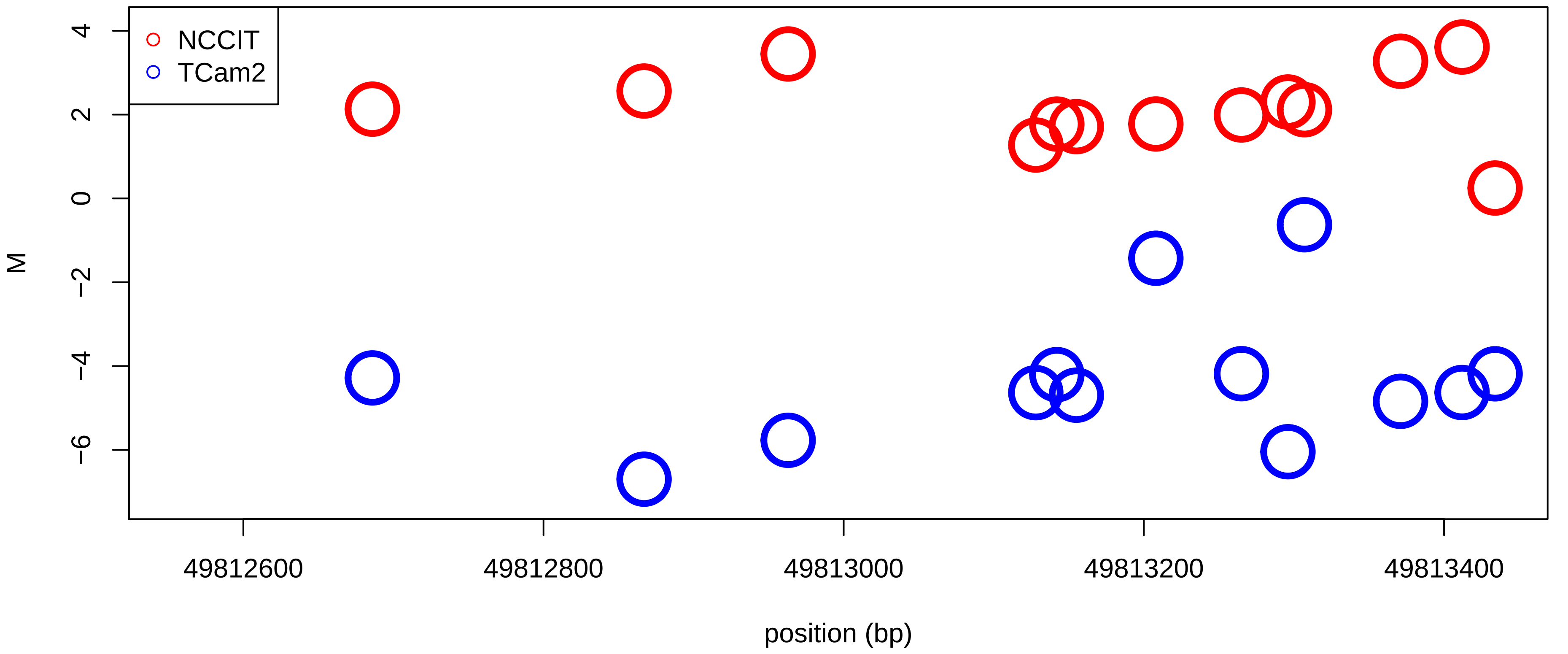

RegionID: 5708, chr10:49812686−49813434−Beta_values

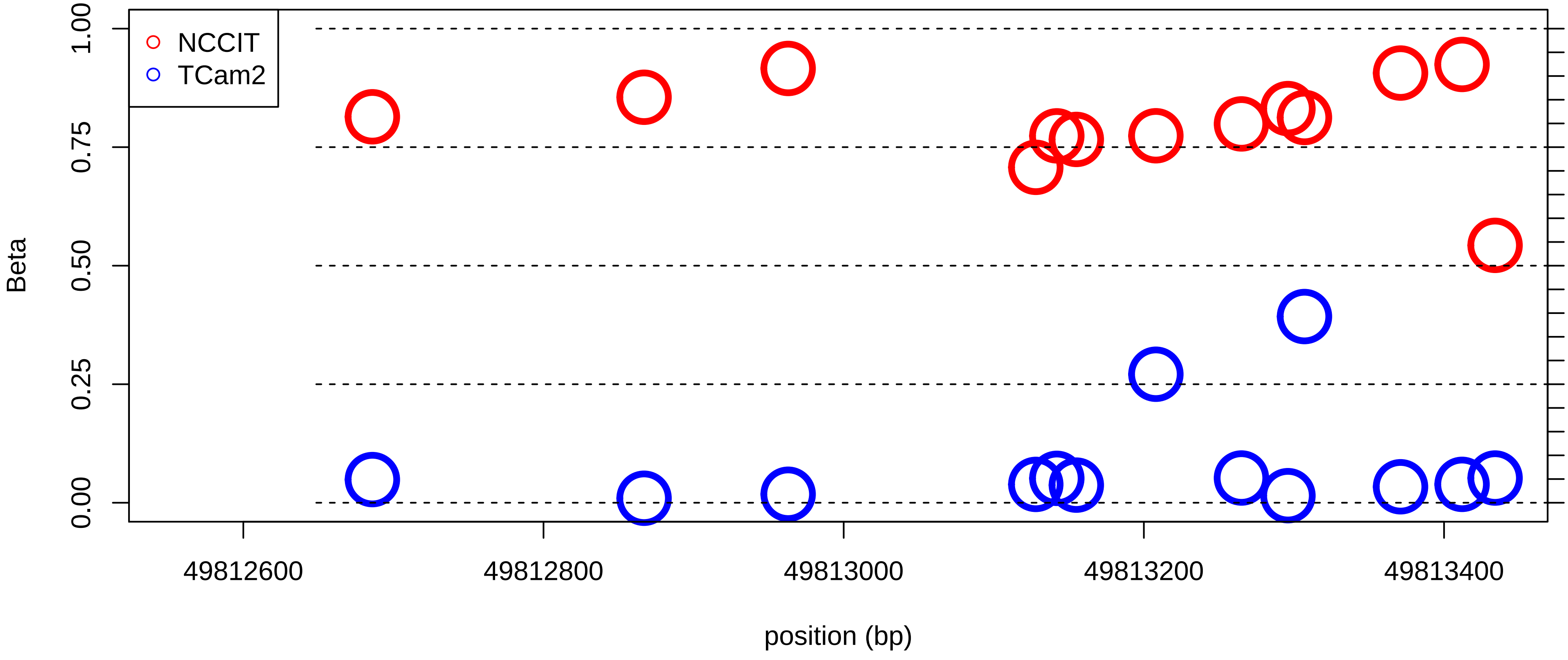

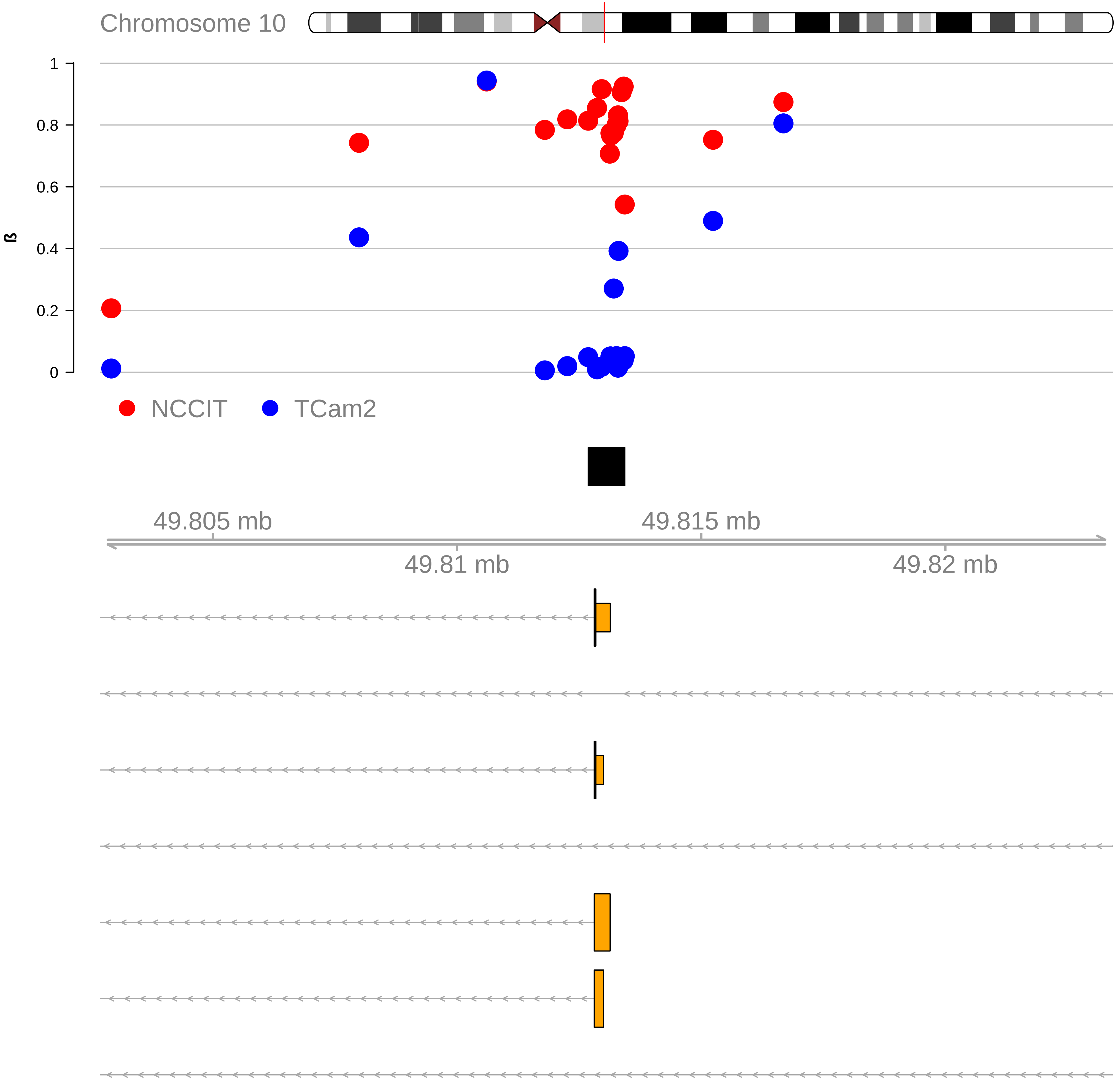

Chromosome 10
β
1
0.8
0.6
0.4
0.2
0
NCCIT
TCam2
49.805 mb
49.815 mb
49.81 mb
49.82 mb
